# Influence of Carboxylate Anions on Phase Behavior of Choline Ionic Liquid Mixtures

**DOI:** 10.3390/molecules25071691

**Published:** 2020-04-07

**Authors:** Fred Elhi, Mikhail Gantman, Gunnar Nurk, Peter S. Schulz, Peter Wasserscheid, Alvo Aabloo, Kaija Põhako-Esko

**Affiliations:** 1 Institute of Technology, University of Tartu, Nooruse 1, 50411 Tartu, Estonia; alvo.aabloo@ut.ee (A.A.); kaija.pohako@ut.ee (K.P.-E.); 2 Forschungszentrum Jülich GmbH, Helmholtz Institute Erlangen-Nürnberg for Renewable Energy (IEK-11), Egerlandstr. 3, 91058 Erlangen, Germany; m.gantman@fz-juelich.de; 3 Institute of Chemistry, University of Tartu, Ravila 14a, 50411 Tartu, Estonia; gunnar.nurk@ut.ee; 4 Department of Chemical and Biological Engineering (CBI), Institute of Chemical Reaction Engineering, University of Erlangen-Nuremberg, Egerlandstraße 3, 91058 Erlangen, Germany; peter.schulz@fau.de (P.S.S.); peter.wasserscheid@fau.de (P.W.)

**Keywords:** choline, eutectic mixture, differential scanning calorimetry, ionic liquid, phase diagram

## Abstract

Mixing ionic liquids is a suitable strategy to tailor properties, e.g., to reduce melting points. The present study aims to widen the application range of low-toxic choline-based ionic liquids by studying eight binary phase diagrams of six different choline carboxylates. Five of them show eutectic points with melting points dropping by 13 to 45 °C. The eutectic mixtures of choline acetate and choline 2-methylbutarate were found to melt at 45 °C, which represents a remarkable melting point depression compared to the pure compounds with melting points of 81 (choline acetate) and 90 °C (choline 2-methylbutarate), respectively. Besides melting points, the thermal stabilities of the choline salt mixtures were investigated to define the thermal operation range for potential practical applications of these mixtures. Typical decomposition temperatures were found between 165 and 207 °C, with choline lactate exhibiting the highest thermal stability.

## 1. Introduction

Ionic liquids (ILs), salts liquid below a generally agreed 100 °C limit, are important in many research fields, such as chemical synthesis, cost efficiency of production, reduction of waste, and toxic reagents. ILs are known for their attractive properties such as high chemical and thermal stability, negligible vapor pressure and interesting electrochemical as well as solvating properties. These properties can be tailored by variation of the composition of cations and anions [1]. Even more variability is offered by using mixtures of ILs, an option that is considered the fourth evolution of ILs [2]. In this contribution, we deal with mixtures of choline carboxylate ILs. We anticipate that these mixtures will widen the application range of this class of ILs. We see potential applications in the medical [3] and environmental fields [1] where these systems offer a combination of low toxicity, adjustable solvation properties, and tunable ionic conductivity [2].

One of the most practically relevant properties of IL mixtures is their low melting point. Given the limited thermal stability of organic ions, a low melting point leads to a wide liquidus range in the resulting ionic system. A viable alternative for low melting ILs and IL mixtures are deep eutectic solvents (DESs) [4], which are defined as eutectic mixtures formed from Lewis or Brønsted acids and bases containing ionic species [5] whose real eutectic point is lower than their ideal eutectic point [6]. Advantages of DESs compared to pure ILs include easy preparation and therefore low production costs, increased biocompatibility in case of certain components, and bigger variety for customization concerning acidity, hydrophobicity, or polarity. DES systems have demonstrated their practical use in several applications, such as interfacial polymerizations [7], electrochemistry [8], and organic reactions [9].

So far, the published research describing IL mixtures has mainly focused on imidazolium [10,11], pyrrolidinium [12], and pyridinium [13] ILs. In these works, significant melting point suppression effects were found for the respective eutectic mixtures. However, imidazolium, pyrrolidinium, and pyridinium ILs and their mixtures face some limitations when it comes to large-scale applications in contact with microorganisms or living matter [14]. Challenges include toxicity aspects and the cost for the organic cation synthesis. This is why a more detailed investigation of choline salts is highly interesting. Based on the available literature, choline salts are characterized by their low toxicity for different cell-lines [15,16], bacteria [17], and other organisms [18]. Moreover, choline salts are industrially available on a large scale.

The most widely studied choline salt used in eutectic mixtures is choline chloride [19,20,21,22,23,24,25,26]. One of the first reported DESs was a mixture of choline chloride (liquefaction temperature 302 °C), and urea (melting point 134 °C) that resulted in a 2:1 molar mixture with a remarkable melting point suppression to 12 °C [4]. The typical strategy was to combine choline ILs with molecular compounds such as carboxylic acids, alcohols, and urea derivates [27], and such choline-based DESs have been used in various applications, e.g., as drug solubilization vehicles [28,29,30]. Note, however, that by mixing a choline IL with molecular substances, some of the very attractive IL properties, e.g., non-volatility, non-flammability, and high ionic conductivity can get lost to a large extent. This is why the mixing of two choline ILs is a very attractive, yet widely unexplored, alternative.

In the present study, we focus on IL mixtures based on choline carboxylates with a different alkyl chain length at the cation, and various anions. The combination of the choline cation with anions derived from carboxylic acids leads to biologically benign ILs, which have been used e.g., in separation processes [31] and microemulsions with low toxicity [32]. As temperature influences the properties of IL mixtures [21,22,25], tailoring the melting point of mixtures opens interesting options for performance optimization. Thus, knowledge of the phase behavior of these IL mixtures is needed to leverage the full application potential of the approach. The purpose of our study is to identify eutectic mixtures of choline carboxylates salts with melting points close to room temperature and high thermal stability. We use differential scanning calorimetry (DSC) to determine the eutectic composition of the mixtures under investigation together with the thermophysical properties of the individual components.

## 2. Results and Discussion

### 2.1. Thermophysical Properties on Choline ILs

Table 1 summarizes the thermophysical information on choline carboxylate mixtures collected in this study. A comparison with previously published data indicates a certain underestimation of the melting points of choline ILs in the literature. For example, the melting point for choline acetate ([Ch][Ac]) measured in the present study (81 °C) is significantly higher than the ones reported previously (51 [33] and 72 °C [34]). For choline isobutyrate ([Ch][Ib]) the difference is even more pronounced: Petkovic et al. reported a melting point of 35 °C [35], while we found 68 °C. The discrepancy in the melting points can be explained by the variable water content and purity of the analyzed samples. The measurement conditions and interpretation of the DSC curves might lead to different results, too: determination of melting points as peak onsets had lower reproducibility compared to the top values reported in our study.

All choline carboxylates showed a glass transition temperature in a narrow range below −60 °C, which is quite typical for ammonium ILs with similar molecular weights [36]. Only a minimal discrepancy between the values from literature and our results was found. For example, we found a glass transition temperature for choline malonate ([Ch][Mal]) of −67 °C, while Mourão, et al. reported −65 °C [31].

Decomposition temperatures were measured to evaluate the thermal stability of the ILs. The Thermogravimetric Analysis (TGA) results for 5% mass decomposition in nitrogen and synthetic air obtained with a heating rate of 10 °C·min^−1^ are presented in Table 1. According to these results, the most stable IL was choline lactate ([Ch][Lac]), and the lowest decomposition temperature was determined for [Ch][Mal], in both inert and oxygen-containing atmospheres. Available literature data on choline salt thermal stability show small deviations from our findings. For [Ch][Ac] we found a decomposition temperature of 178 °C, compared with 189 °C reported by Fukaya et al. [33]. For [Ch][Ib], we reported 178 °C, compared with 172 °C reported by Petkovic et al. [35]. These differences reflect variabilities in the sample quality and the measurement protocols, e.g., concerning the applied heating ramp.

Interestingly, we found only a minor difference in decomposition temperatures between inert conditions and the oxygen containing atmosphere in our TGA experiments. The exception was [Ch][Mal], which showed a significantly lower decomposition temperature in synthetic air as compared with nitrogen. Taking into account that TGA measurements with a heating rate of 10 °C·min^−1^ tend to overestimate thermal stability [37], we conclude that the other choline ILs under investigation show at least short-term stability, even in oxygenated environments at temperatures up to 170 °C in these particular dynamic conditions.

### 2.2. Thermophyscial Behaviour of the IL Mixtures

Overall, we report eight phase diagrams involving six different ILs as mixtures. First, [Ch][Ac] was combined with other choline salts. ILs that gave eutectic mixtures with [Ch][Ac], namely [Ch][Ib], choline isovalerate ([Ch][Iv]), and choline 2-methylbutyrate ([Ch][2mb]), were subsequently combined with each other (Table 2). The binary mixtures with [Ch][Ac] could be divided into three categories: mixtures with monocarboxylic ILs showed eutectic behavior, mixtures with [Ch][Mal] resulted in gradually lower melting points with an increasing concentration of [Ch][Mal], and finally, mixtures with [Ch][Lac] did not show crystallization in our study. All studied mixtures showed glass transition temperatures very close to those of the pure ILs.

**Figure 1:**
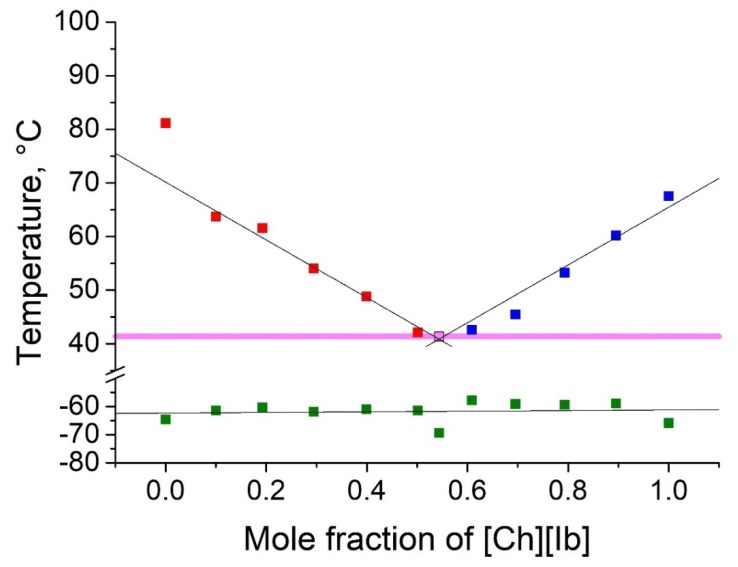
Temperature composition phase diagram for the mixture of choline acetate ([Ch][Ac]) and choline isobutyrate ([Ch][Ib]), and their individual components at 0.0 and 1.0 mole fractions. Legend: ▪—melting point of the excess component [Ch][Ac] in the mixture; ▪—melting point of the excess component [Ch][Ib] in the mixture; ▪—glass transition temperature; ▪—eutectic point; – eutectic transition line.

**Figure 2:**
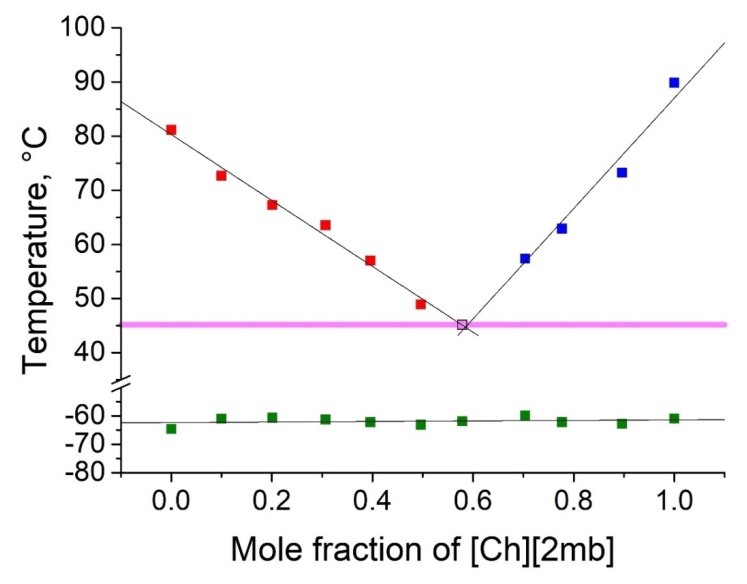
Temperature composition phase diagram for the mixture of [Ch][Ac] and choline 2-methylbutyrate ([Ch][2mb]), and their individual components at 0.0 and 1.0 mole fractions. Legend: ▪—melting point of the excess component [Ch][Ac] in the mixture; ▪—melting point of the excess component [Ch][2mb] in the mixture; ▪—glass transition temperature; ▪—eutectic point; – eutectic transition line.

**Figure 3:**
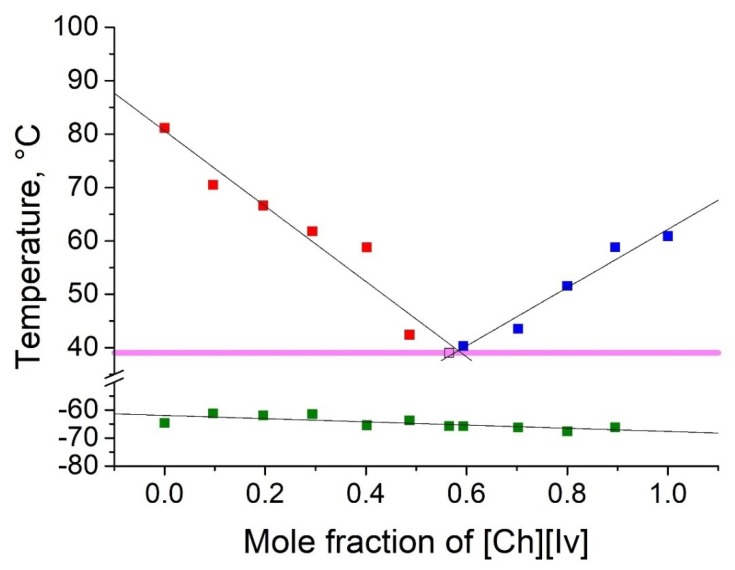
Temperature composition phase diagram for the mixture of [Ch][Ac] and choline isovalerate ([Ch][Iv]), and their individual components at 0.0 and 1.0 mole fractions. Legend: ▪—melting point of the excess component [Ch][Ac] in the mixture; ▪—melting point of the excess component [Ch][Iv] in the mixture; ▪—glass transition temperature; ▪—eutectic point; – eutectic transition line.

**Figure 4:**
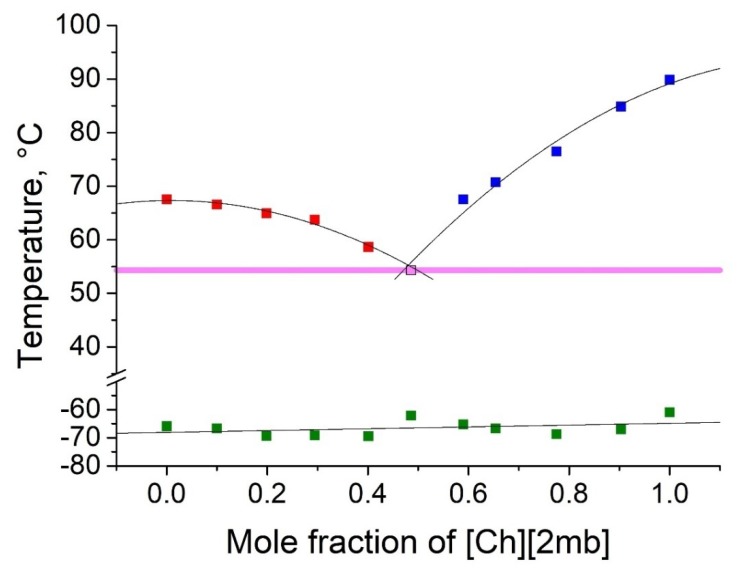
Temperature-composition phase diagram for the mixture of [Ch][Ib] and [Ch][2mb], and their individual components at 0.0 and 1.0 mole fractions. Legend: ▪—melting point of the excess component [Ch][Ib] in the mixture; ▪—melting point of the excess component [Ch][2mb] in the mixture; ▪—glass transition temperature; ▪—eutectic point; – eutectic transition line.

**Figure 5:**
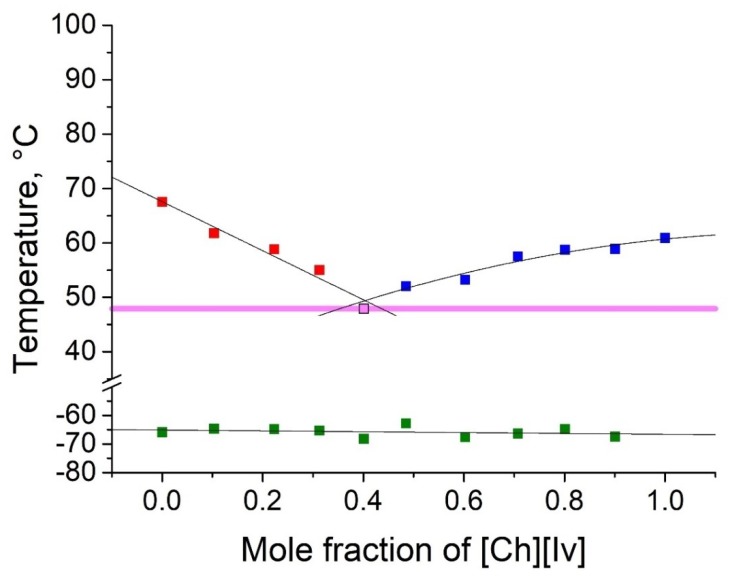
Temperature-composition phase diagram for the mixture of [Ch][Ib] and [Ch][Iv], and their individual components at 0.0 and 1.0 mole fractions. Legend: ▪—melting point of the excess component [Ch][Ib] in the mixture; ▪—melting point of the excess component [Ch][Iv] in the mixture; ▪—glass transition temperature; ▪—eutectic point; – eutectic transition line.

As shown in Table 2, five binary eutectic mixtures were found. The corresponding phase diagrams are presented as Figure 1, Figure 2, Figure 3, Figure 4 and Figure 5, and the Tammann plots are shown in the Supplementary Materials. Unfortunately, none of the eutectic mixtures were liquid at room temperature (Figure 1, Figure 2, Figure 3, Figure 4 and Figure 5, Table 3). The lowest melting point achieved was found for the eutectic mixture of [Ch][Ac] and [Ch][Iv], for which a melting temperature of 39 °C was determined. All eutectic points were found at a molar fraction between 0.6 and 0.4 for both components. The greatest melting point depression of 45 °C (compared to the pure ILs) was determined for the case of the [Ch][2mb] + [Ch][Ac] mixture.

Low IL melting points can be achieved when pairing ions with different levels of bulkiness. Extending this idea to mixtures helps to understand the melting point depression observed. A comparison of the obtained eutectic mixtures (Table 3) revealed that mixing anions with different sizes, like [Ac]^−^ and [2mb]^−^, leads to a somewhat greater melting point depression than mixing anions of similar levels of bulkiness, such as [Iv]^−^ and [2mb]^−^.

**Figure 6:**
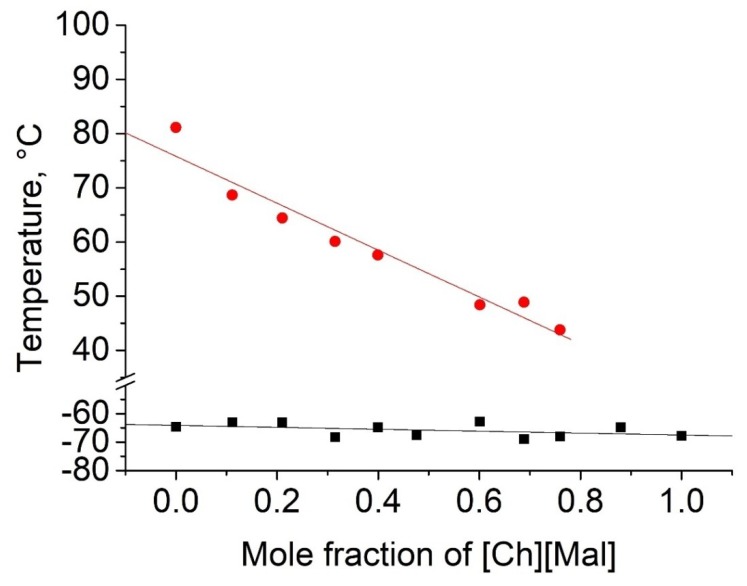
Temperature-composition phase diagram for the mixture of [Ch][Ac] and choline malonate ([Ch][Mal]). Legend: ●—melting points of mixture and [Ch][Ac] component at 1.0 mole fraction; ▪—glass transition temperatures of the mixture and [Ch][Ac] component at 1.0 mole fraction.

Three of the studied binary phase diagrams did not present eutectic behavior (Table 3). Mixtures of [Ch][Ac] and [Ch][Mal] exhibited melting point decreases with an increasing concentration of [Ch][Mal] until a mole fraction of 0.8 was reached. Further addition of [Ch][Mal] resulted in mixtures with only glass transition behavior (Figure 6). In contrast, when choline chloride was mixed with malonic acid, a eutectic mixture was formed at 50 mol% [38]. Mixtures with no detectable melting point have been called low-transition mixtures [39], and a lack of crystallization has been also related to the high viscosity of these mixtures [28]. It is worth mentioning that the absence of melting or freezing in the DSC measurement does not indicate that these phase transitions are impossible. It simply means that crystallization did not occur under the applied conditions of our experiment.

Exothermic devitrification effects were often detected in the melting curves of the DSC thermograms (a typical example is presented in the Supplementary Materials). These were interpreted as solid–solid crystallization effects, where glass crystallizes into a solid and later melts at a higher temperature [28,40]. The presence of several exothermic transitions indicates the existence of different crystal structures at different temperatures [41]. So-called cold crystallization (when a low-temperature metastable state turns into a stable crystalline state, which only happens during heating) is highly dependent on the heating rate [41,42]. Such effects have also been described previously for pyrrolidinium [43], imidazolium [41], and pyridinium [13] ILs, and for binary IL mixtures [44].

## 3. Materials and Methods

### 3.1. Materials

The tested choline ILs were synthesized by mixing choline bicarbonate (Sigma-Aldrich, St. Louis, USA, 80% in H_2_O) with carboxylic acids (acetic acid, lactic acid, malonic acid, isovaleric acid, isobutyric acid, 2-methylbutyric acid; all from Sigma-Aldrich, used as received) in aqueous solutions [34]. The chemical identity of the obtained ILs (for structures, names and abbreviations, see Table 1) was confirmed by ^1^H-NMR spectroscopy (Bruker Avance II 400 MHz, Bruker Corporation, Billerica, MA, USA) using D_2_O as a solvent. The water content was determined by Karl Fischer’s coulometric titration (Metrohm 756 KF Coulometer, Metrohm AG, Herisau, Switzerland). All ILs were dried under high vacuum and stored in a glovebox under an argon atmosphere (water content below 0.5 ppm) prior to use.

### 3.2. DSC Sample Preparation

For binary IL mixtures consisting of components A and B, the molar concentrations of both components varied in the range from 100% to 0% with 10% steps. The IL mixtures were prepared by weighing the appropriate amounts of components A and B. The mixture was stirred at 100 °C under argon to ensure homogeneity. They crystallized upon cooling down. For DSC measurements, 2–9 mg of the IL mixtures or of the pure ILs were weighted into aluminum sample pans. All sample handling was carried out under an Ar atmosphere. DSC samples were prepared immediately before the measurement.

### 3.3. DSC Measurements

The DSC measurements were performed using a NETZSCH DSC 204 F1 Phoenix^®^ differential scanning calorimeter (NETZSCH Group, Selb, Germany) equipped with nitrogen cooling accessories, under normal pressure (p = 1 bar). The measurements were carried out in three repeated cycles in the temperature range −100 to 100 °C (except for mixtures with [Ch][Iv], which were measured from −50 to 150 °C). The samples were cooled to −100 °C at a screening rate of 10 °C·min^−1^ and kept at an isotherm for 30 min to facilitate crystallization (3 h in case of [Ch][Mal] and [Ch][Lac]), then heated with a temperature ramp of 5 °C·min^−1^ to 100 °C. An empty sample pan with a small hole in the lid served as the reference. To avoid condensation of water, the instrument was purged with dry nitrogen.

Top values of phase transition peaks during heating in the second cycle are reported as melting points. The uncertainty of temperatures was estimated to be 1 °C. The phase transition of the heating curve should be preferred because, unlike crystallization, it is not dependent on the history of the sample and the measurement conditions. For each binary system, eleven different concentrations were measured. Based on these data, the Tammann plots (Supplementary Materials) and the liquidus lines of the phase diagrams (Figure 1, Figure 2, Figure 3, Figure 4 and Figure 5) were constructed, using linear and second order polynomial fits (OriginPro 9.1 inbuilt function). Eutectic compositions were found from the Tammann plots and additional DSC measurements were carried out with the supposed eutectic mixtures to experimentally confirm their melting points. For clear determination of the possible phases at each composition of the mixtures tested, the eutectic thermal transitions for the mixtures tested are depicted as eutectic transition lines in Figure 1, Figure 2, Figure 3, Figure 4 and Figure 5. Glass transitions temperatures were determined for all samples as the midpoint of the glass transition part of the DSC curve.

### 3.4. TGA Measurements

Thermogravimetric analyses (TGA, NETZSCH STA449F3, Selb, Germany) were performed to determine the decomposition temperatures of the ILs under investigation. Samples were placed in Al_2_O_3_ crucibles and heated from 40 to 300 °C with a heating rate of 10 °C·min^−1^ under a constant flow of N_2_ or synthetic air (80 vol% N_2_ and 20 vol% O_2_). The 5% mass loss temperature values were taken from the corresponding inflection point on the thermogravimetric (TG) curve where the necessary mass loss was detected. The data obtained do not represent the long-term stability of the investigated IL mixtures in application scenarios. They rather reflect relative stability differences among the mixtures under investigation.

## 4. Conclusions

The phase behavior of binary mixtures of choline carboxylate ILs has been investigated. Eight phase diagrams have been reported from mixtures of six different choline carboxylates. Five of the obtained phase diagrams presented eutectic points with melting point suppression between 13 and 45 °C. Anions with different levels of bulkiness like, for example, in the mixture of [Ch][Ac] and [Ch][2mb], led to a higher decrease in the melting point at the eutectic composition compared to anions of similar size, like in [Ch][Iv] and [Ch][2mb]. More drastic changes in the anion structure, such as the addition of carboxylate or hydroxyl group in the case of [Ch][Mal] and [Ch][Lac] resulted in mixtures without eutectic behavior.

From the thermogravimetric analysis, we conclude that the tested choline ILs can be applied at temperatures below 100 °C under inert as well as under oxygen-containing atmospheres. The thermal stability measurements with a steep heating rate of 10 °C·min^−1^ gave decomposition temperatures between 129 and 206 °C with a clear dependence on the IL anion. Choline lactate was found to be the most stable salt among the tested choline salts.

The present study adds useful data for the wider applications of choline salts and their mixtures. As many choline salts are solid at ambient temperature, their usage as low-toxicity alternatives for conventional ILs has been limited so far. Our study proves that mixing different choline salts can lead to significantly lower melting points while fully maintaining the typical IL properties of extremely low vapor pressure and high ionic conductivity. Excellent availability and low toxicity of both the choline cation and the short-chain carboxylate anions make the here-described choline salt mixtures interesting candidates for solvent applications in the food and pharmaceutical industry.

## Figures and Tables

**Table 1:** Key properties of tested ionic liquids (ILs).

IL	Abbreviation	Anion Structure	T_g_	T_m_	*w*%	T_d, 5%, N2_	T_d, 5%, air_
choline acetate	[Ch][Ac]	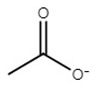	−65	81	2.7	178	176
choline isobutyrate	[Ch][Ib]	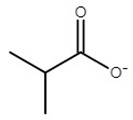	−66	68	0.1	178	172
choline isovalerate	[Ch][Iv]	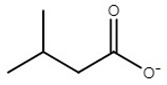	NA*	61	0.0	176	176
choline 2-methylbutyrate	[Ch][2mb]	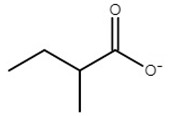	−61	90	0.3	175	174
choline malonate	[Ch][Mal]	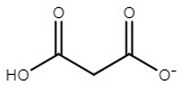	−67	ND**	0.2	165	129
choline lactate	[Ch][Lac]	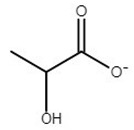	−62	ND**	0.8	207	206

T_g_—glass transition temperature in °C; T_m_—melting temperature in °C; *w*%—water content in %; T_d, 5%, N2_—5% mass loss temperature in 100% N_2_ atmosphere in °C; T_d, 5%, air_—5% mass loss temperature in 80% N_2_ and 20% O_2_ atmosphere in °C; * NA—not available; ** ND—not detected in this study.

**Table 2:** Overview of the studied binary phase diagrams. (Shading provided to avoid double entries and non-mixtures.)

	[Ch][Ac]	[Ch][Ib]	[Ch][Iv]	[Ch][2mb]	[Ch][Mal]	[Ch][Lac]
**[Ch][Ac]**						
**[Ch][Ib]**	eutectic, T_m_, T_g_					
**[Ch][Iv]**	eutectic, T_m_, T_g_	eutectic, T_m_, T_g_				
**[Ch][2mb]**	eutectic, T_m_, T_g_	eutectic, T_m_, T_g_	T_m_, T_g_			
**[Ch][Mal]**	T_m_, T_g_	-	-	-		
**[Ch][Lac]**	T_g_	-	-	-	-	

**Table 3:** Properties of eutectic compositions of choline ILs in this study.

Mixture			Eutectic Composition		Melting Point Depression	
Comp. A	Comp. B	T_m, eu_, °C	Comp. A	Comp. B	T_m,A_, °C	T_m,B_, °C
**[Ch][Ac]**	**[Ch][Ib]**	41	0.46	0.54	40	26
**[Ch][Ac]**	**[Ch][2mb]**	45	0.42	0.58	36	45
**[Ch][Ac]**	**[Ch][Iv]**	39	0.43	0.57	42	22
**[Ch][Ib]**	**[Ch][2mb]**	54	0.51	0.49	13	36
**[Ch][Ib]**	**[Ch][Iv]**	48	0.60	0.40	20	13

T_m, eu_—melting point of eutectic mixture; T_m,A_—melting point of pure component A; T_m,B_—melting point of pure component B.

## Supplementary Materials

The following are available online at https://www.mdpi.com/1420-3049/25/7/1691/s1, Figure S1: Tammann diagram of the mixture from [Ch][Ac] + [Ch][Ib], Figure S2: Tammann diagram of the mixture from [Ch][Ac] + [Ch][2mb], Figure S3: Tammann diagram of the mixture from [Ch][Ac] + [Ch][Iv], Figure S4: Tammann diagram of the mixture from [Ch][Ib] + [Ch][2mb], Figure S5: Tammann diagram of the mixture from [Ch][Ib] + [Ch][Iv], Figure S6: DSC thermogram of [Ch][Ac] (39.1%) and [Ch][Ib] (60.9%) mixture showing the cold-crystallization peak at −34°C, Figure S7: An example of a DSC thermogram of [Ch][Ac] (0.46) and [Ch][Ib] (0.54) eutectic mixture, Figure S8: An example of a DSC thermogram of [Ch][Ac] (0.42) and [Ch][2mb] (0.58) eutectic mixture, Figure S9: An example of a DSC thermogram of [Ch][Ac] (0.43) and [Ch][Iv] (0.57) eutectic mixture, Figure S10: An example of a DSC thermogram of [Ch][Ib] (0.51) and [Ch][2mb] (0.49) eutectic mixture, Figure S11: An example of a DSC thermogram of [Ch][Ib] (0.60) and [Ch][Iv] (0.40) eutectic mixture, Figure S12: The DSC diagram of [Ch][Ac], Figure S13: The DSC diagram of [Ch][Ib], Figure S14: The DSC diagram of [Ch][Iv], Figure S15: The DSC diagram of [Ch][Mal], Figure S16: The DSC diagram of [Ch][Lac], Figure S17: The ^1^H NMR of [Ch][Ac], Figure S18: The ^1^H NMR of [Ch][Ib], Figure S19: The ^1^H NMR of [Ch][Iv], Figure S20: The ^1^H NMR of [Ch][2mb], Figure S21: The ^1^H NMR of [Ch][Mal], Figure S22: The ^1^H NMR of [Ch][Lac], Figure S23: The ^13^C NMR of [Ch][Mal].
