# Supplementary material for: Influence of Carboxylate Anions on Phase Behavior of Choline Ionic Liquid Mixtures
Source: Molecules. 2020 Apr 7;25(7):1691. doi: 10.3390/molecules25071691 (PMC7180831; doi:10.3390/molecules25071691)
Supplement: Supplementary file 1 [file molecules-25-01691-s001.pdf]

molecules

Supplementary Materials

Influence of Carboxylate Anions on Phase Behavior of Choline Ionic Liquid Mixtures

Fred Elhi [1,*], Mikhail Gantman [2], Gunnar Nurk [3], Peter S. Schulz [4], Peter Wasserscheid [4], Alvo Aabloo [1] and Kaija Põhako-Esko [1]

1 Institute of Technology, University of Tartu, Nooruse 1, 50411 Tartu, Estonia; alvo.aabloo@ut.ee (A.A.); kaija.pohako@ut.ee (K.P.-E.)
2 Forschungszentrum Jülich GmbH, Helmholtz Institute Erlangen-Nürnberg for Renewable Energy (IEK-11), Egerlandstr. 3, 91058 Erlangen, Germany; m.gantman@fz-juelich.de
3 Institute of Chemistry, University of Tartu, Ravila 14a, 50411 Tartu, Estonia; gunnar.nurk@ut.ee
4 Department of Chemical and Biological Engineering (CBI), Institute of Chemical Reaction Engineering, University of Erlangen-Nuremberg, Egerlandstraße 3, 91058 Erlangen, Germany; peter.schulz@fau.de (P.S.S.); peter.wasserscheid@fau.de (P.W.)
* Correspondence: fred.elhi@ut.ee; Tel.: +372 533 20 474

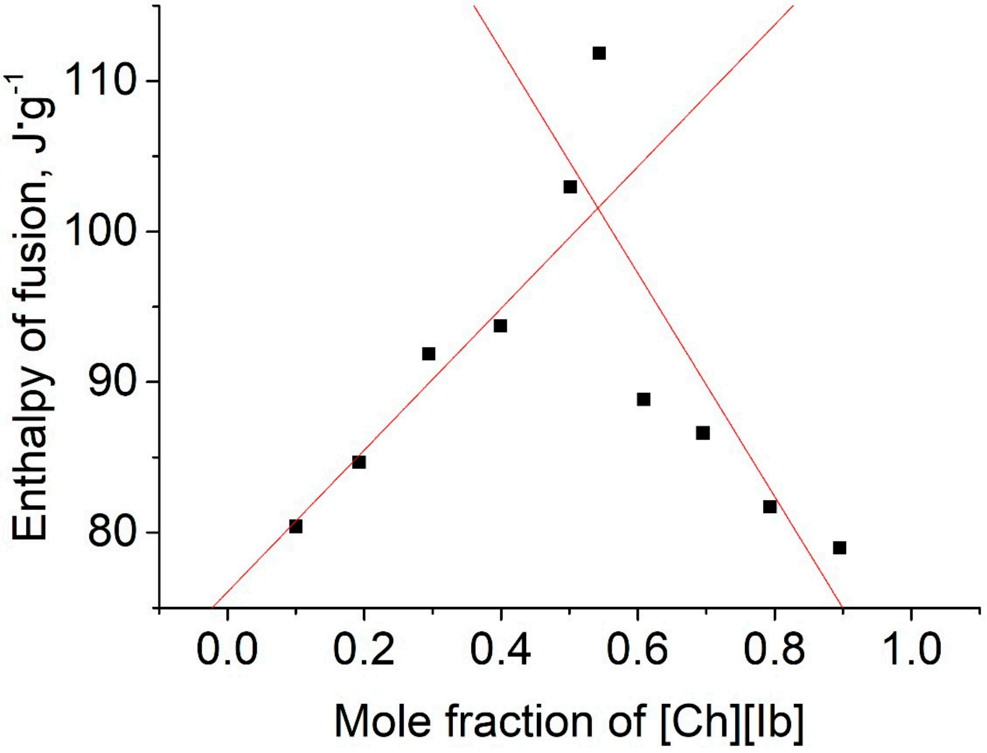

Figure S1. Tammann diagram of the mixture from [Ch][Ac] + [Ch][Ib].

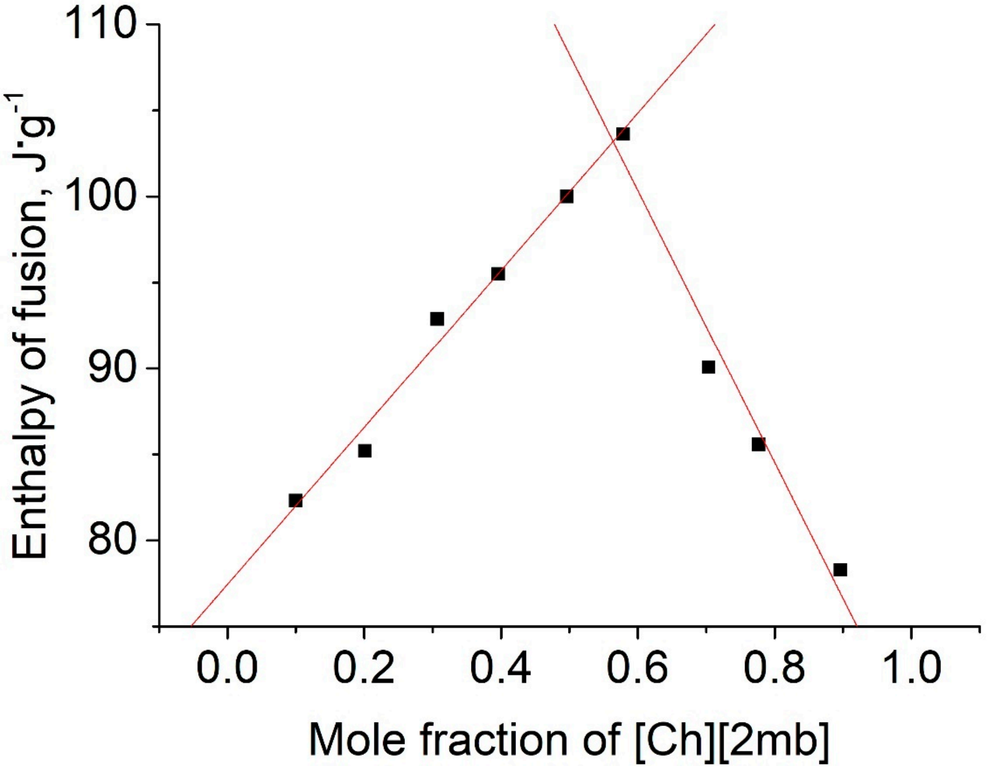

Figure S2. Tammann diagram of the mixture from [Ch][Ac] + [Ch][2mb].

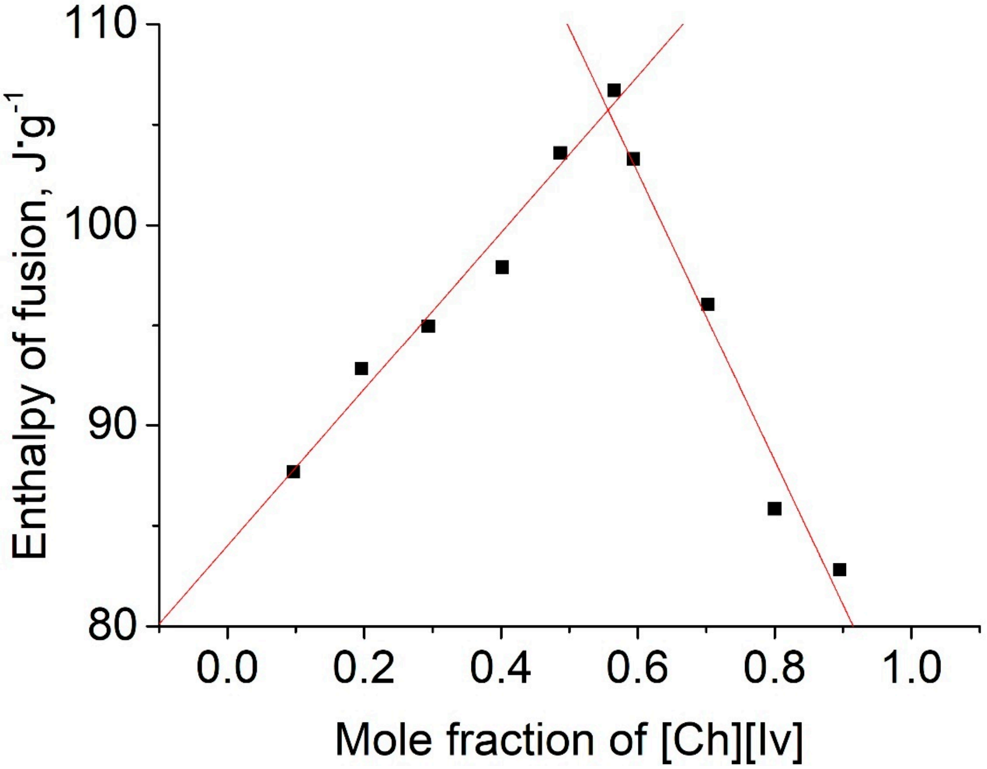

Figure S3. Tammann diagram of the mixture from [Ch][Ac] + [Ch][Iv].

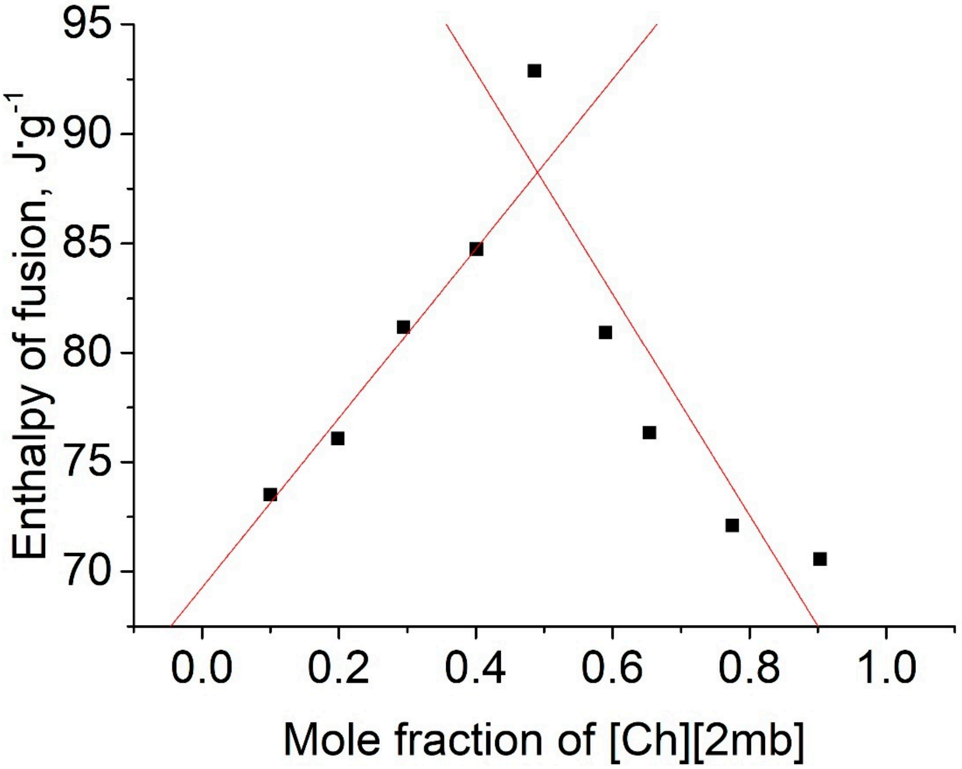

Figure S4. Tammann diagram of the mixture from [Ch][Ib] + [Ch][2mb].

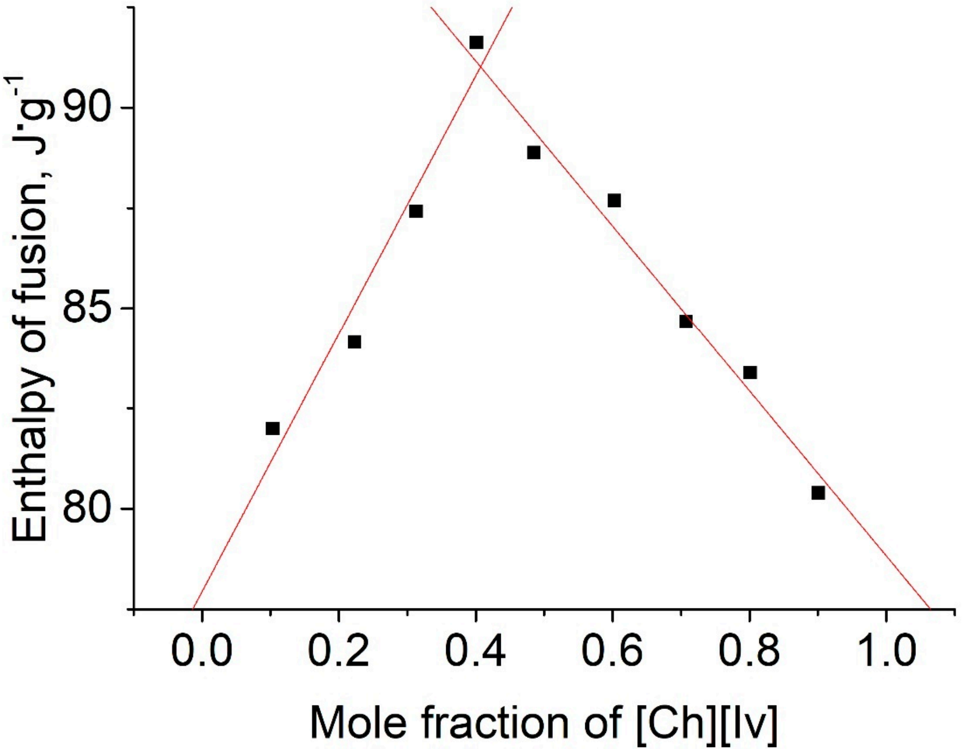

Figure S5. Tammann diagram of the mixture from [Ch][Ib] + [Ch][Iv].

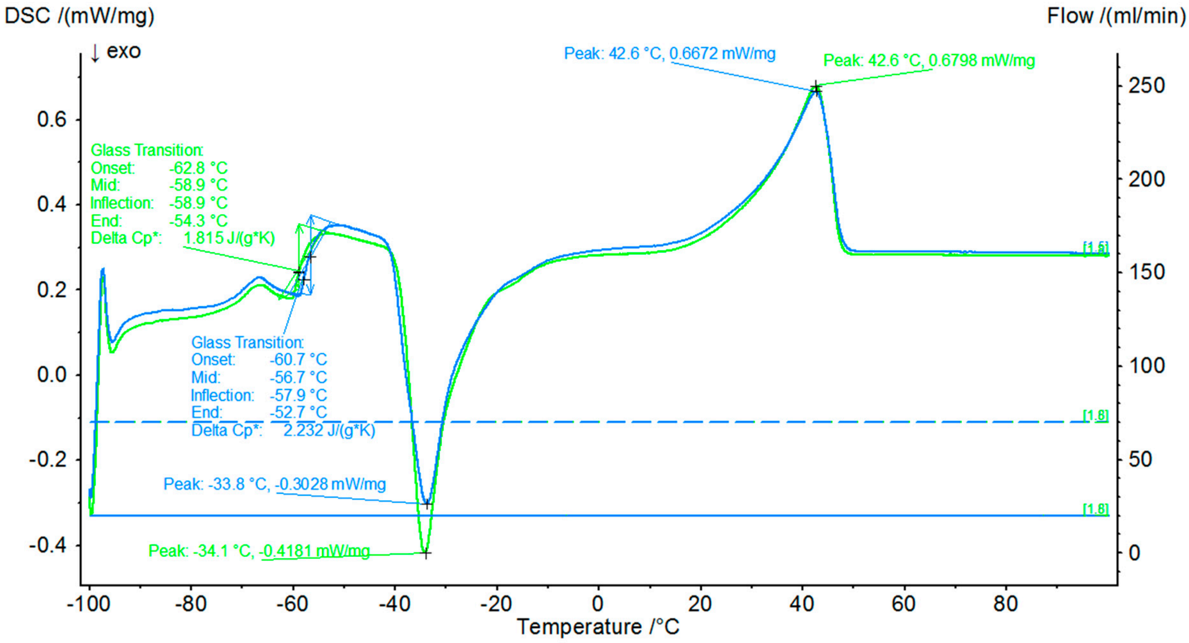

Figure S6. DSC thermogram of [Ch][Ac] (39.1%) and [Ch][Ib] (60.9%) mixture showing the cold-crystallization peak at -34°C.

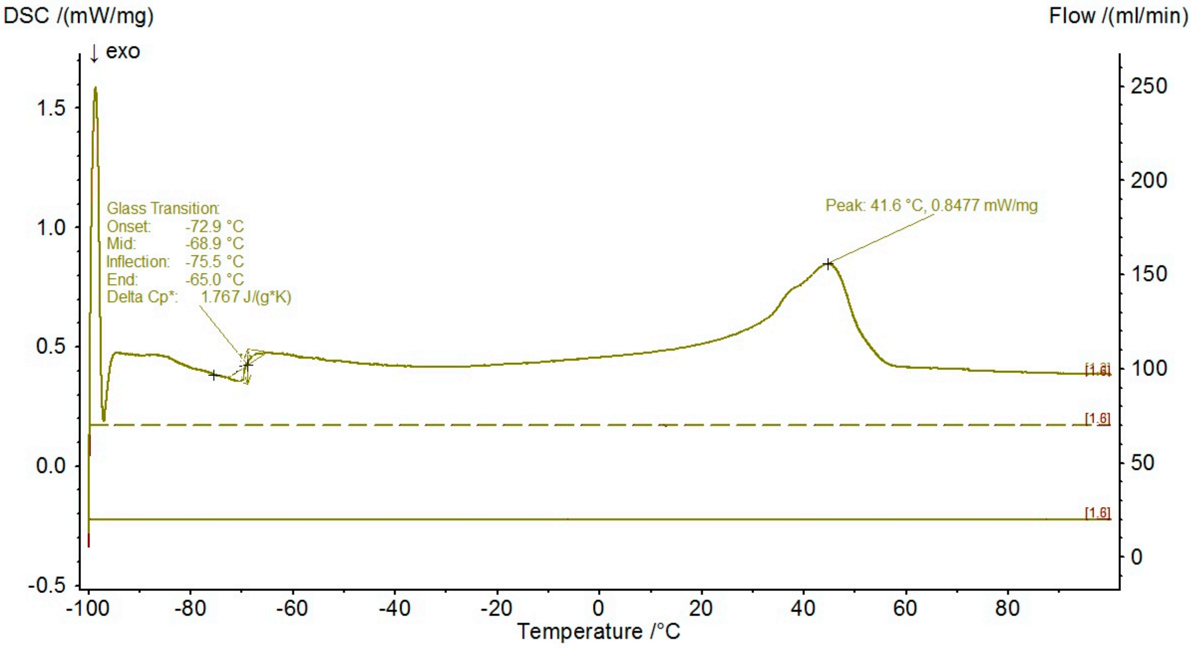

Figure S7. An example of a DSC thermogram of [Ch][Ac] (0.46) and [Ch][Ib] (0.54) eutectic mixture.

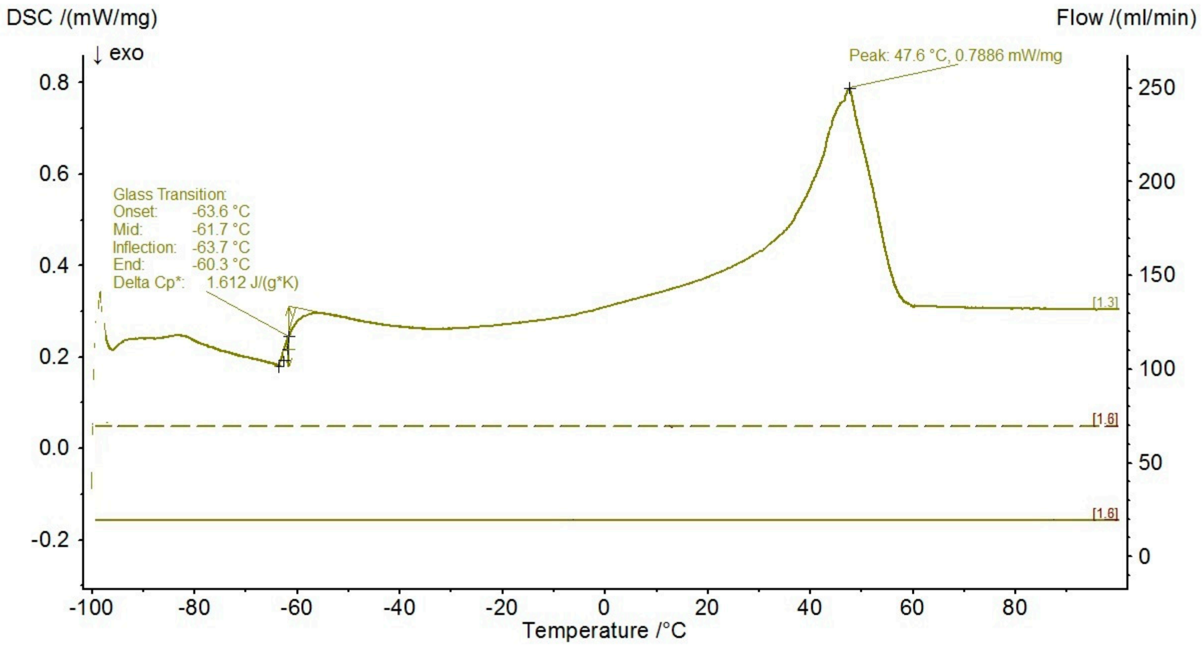

Figure S8. An example of a DSC thermogram of [Ch][Ac] (0.42) and [Ch][2mb] (0.58) eutectic mixture

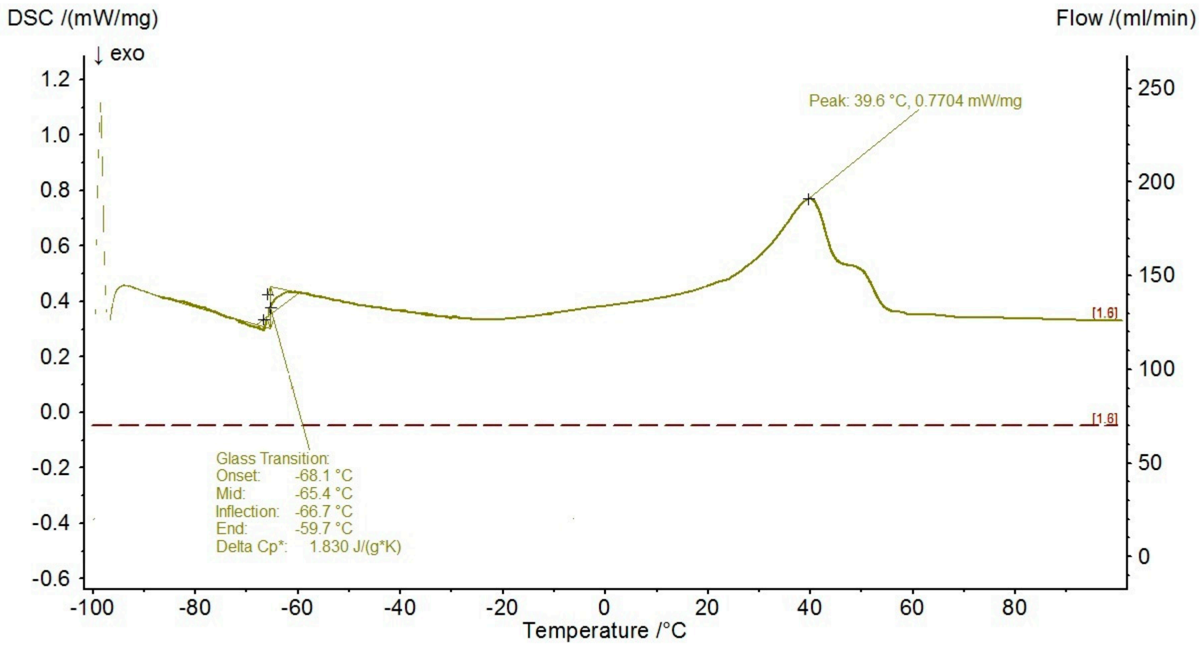

Figure S9. An example of a DSC thermogram of [Ch][Ac] (0.43) and [Ch][Iv] (0.57) eutectic mixture

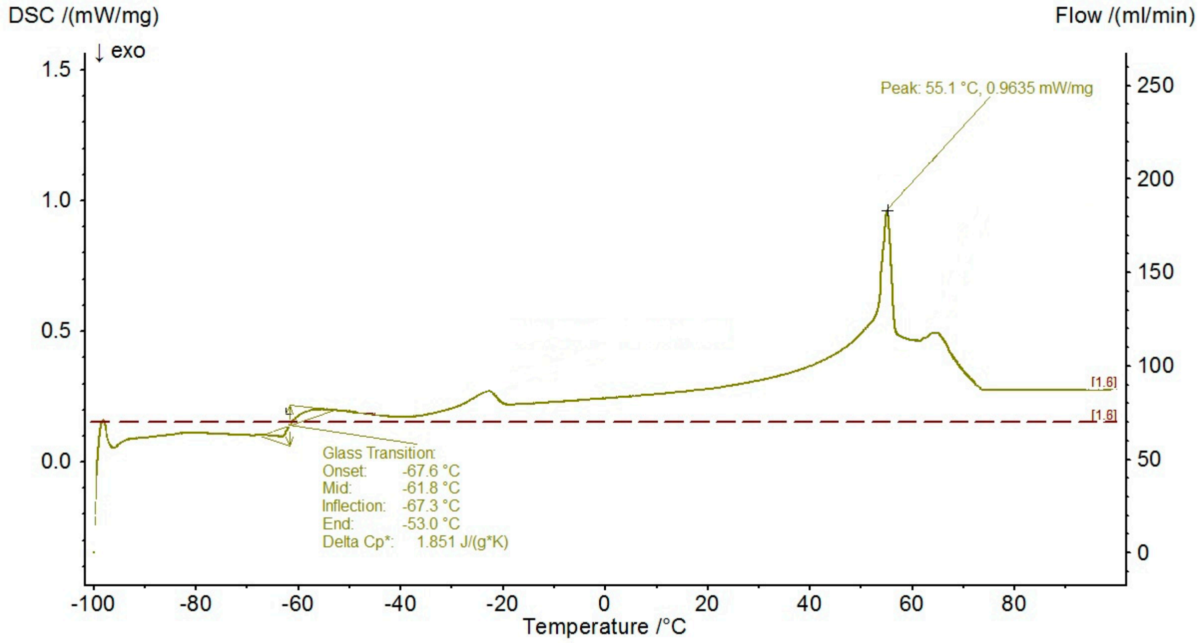

Figure S10. An example of a DSC thermogram of [Ch][Ib] (0.51) and [Ch][2mb] (0.49) eutectic mixture

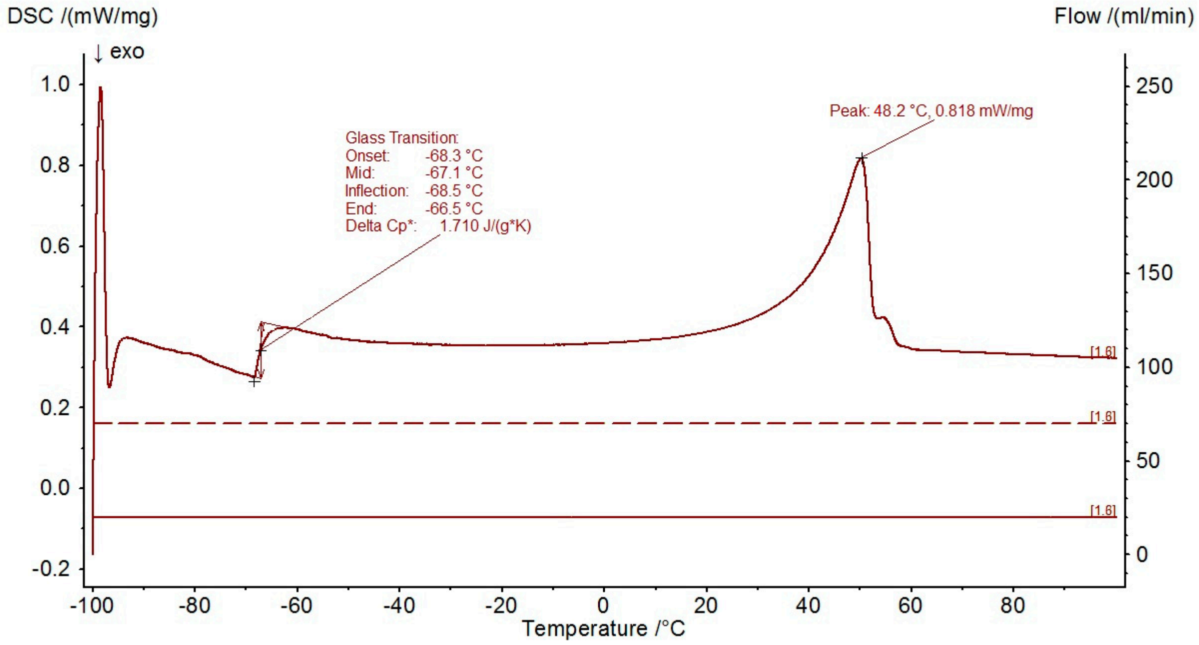

Figure S11. An example of a DSC thermogram of [Ch][Ib] (0.60) and [Ch][Iv] (0.40) eutectic mixture

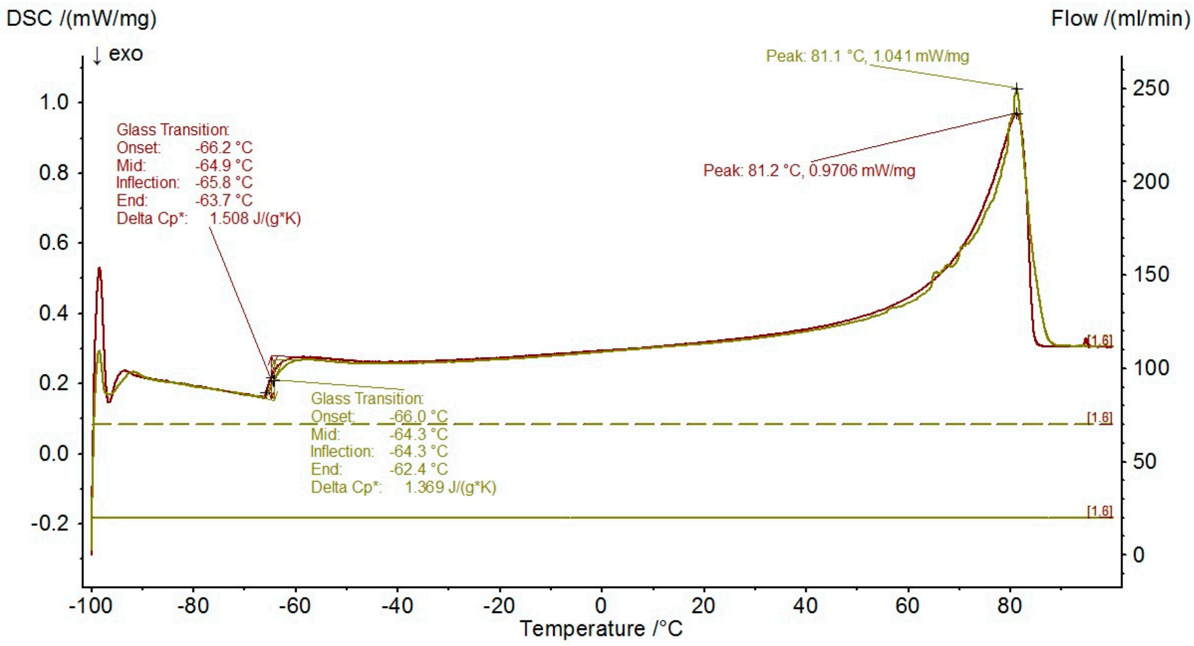

Figure S12. The DSC diagram of [Ch][Ac].

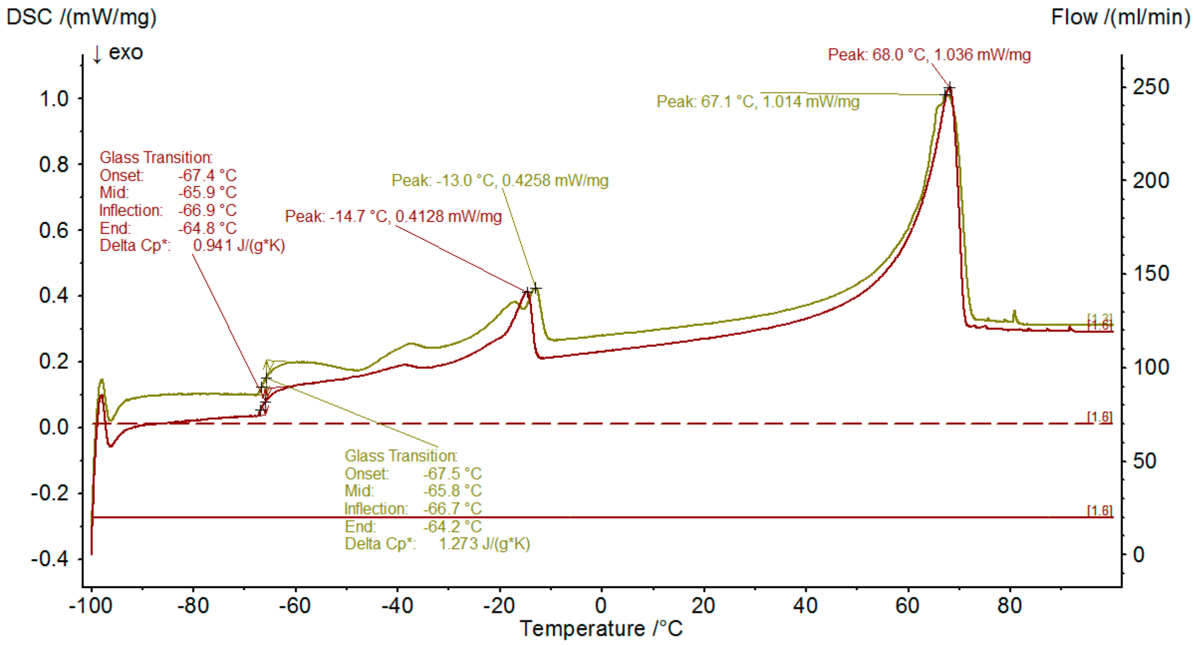

Figure S13. The DSC diagram of [Ch][Ib].

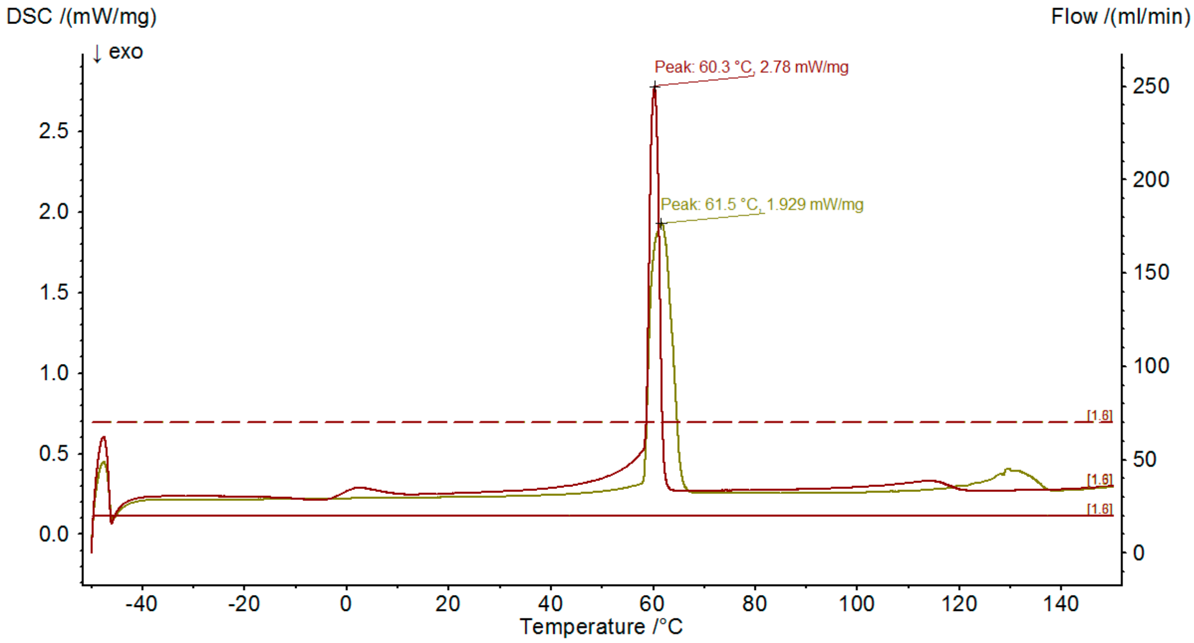

Figure S14. The DSC diagram of [Ch][Iv].

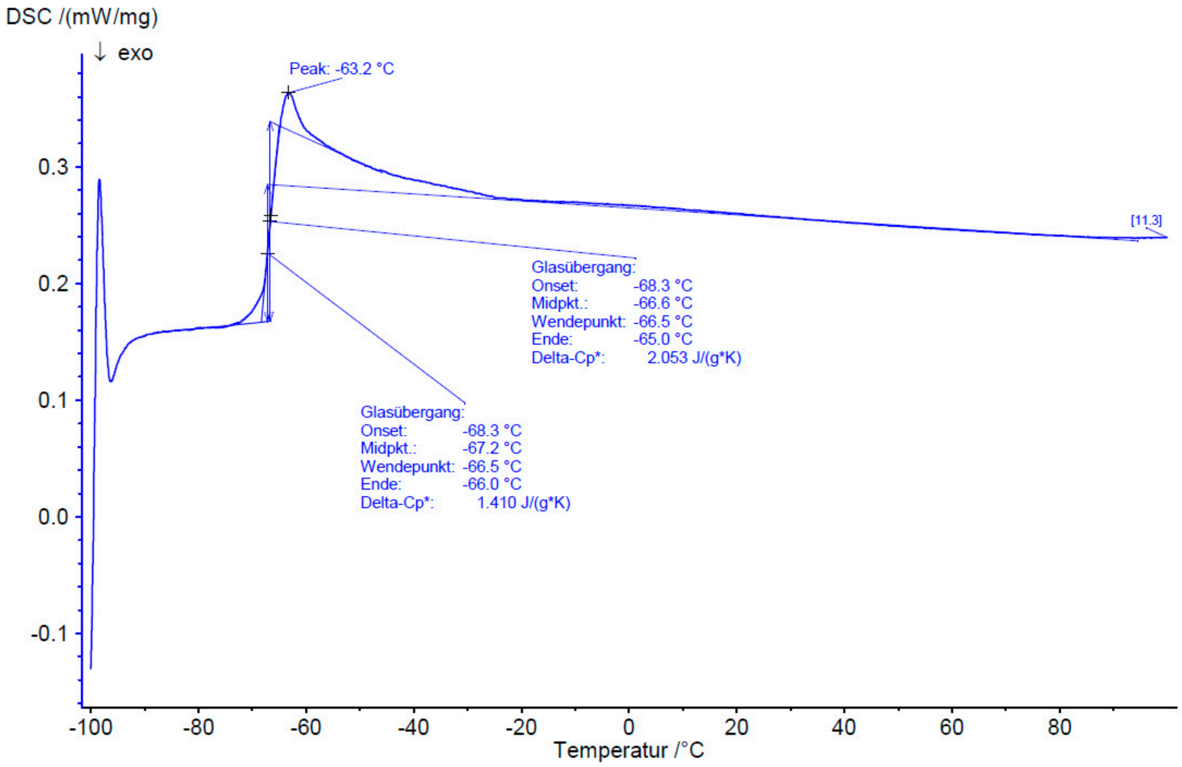

Figure S15. The DSC diagram of [Ch][Mal].

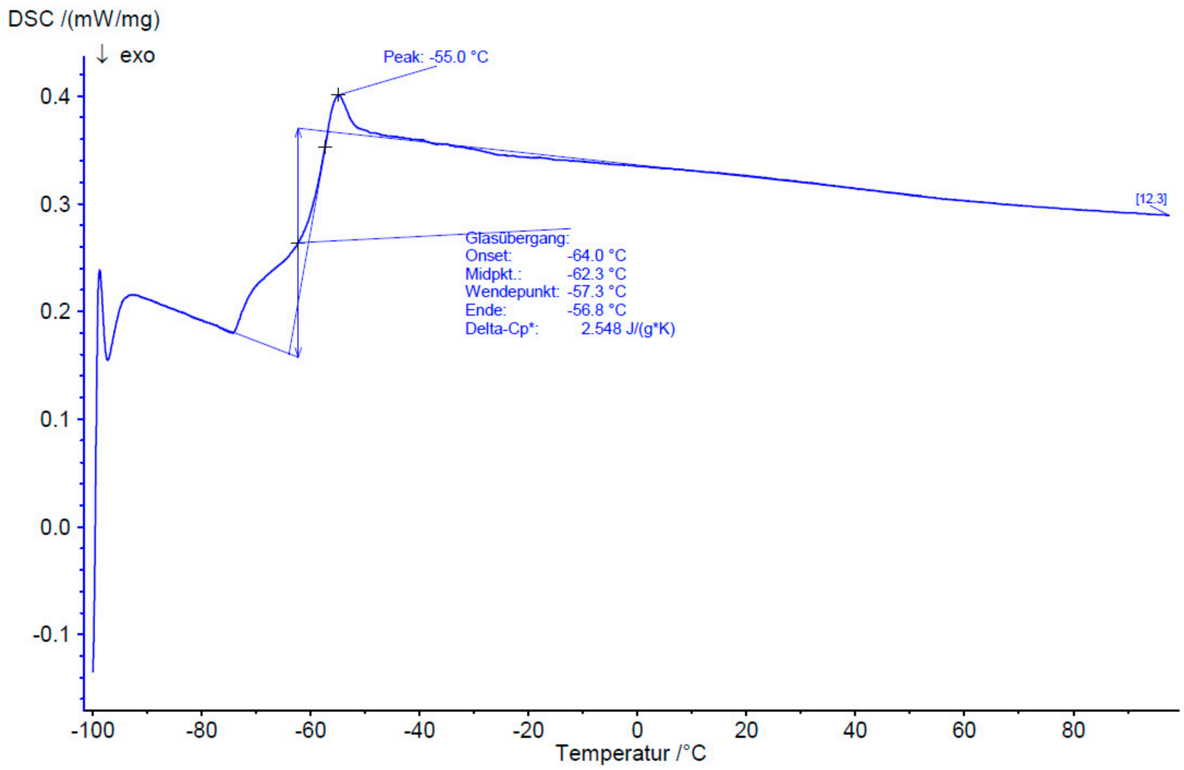

Figure S16. The DSC diagram of [Ch][Lac].

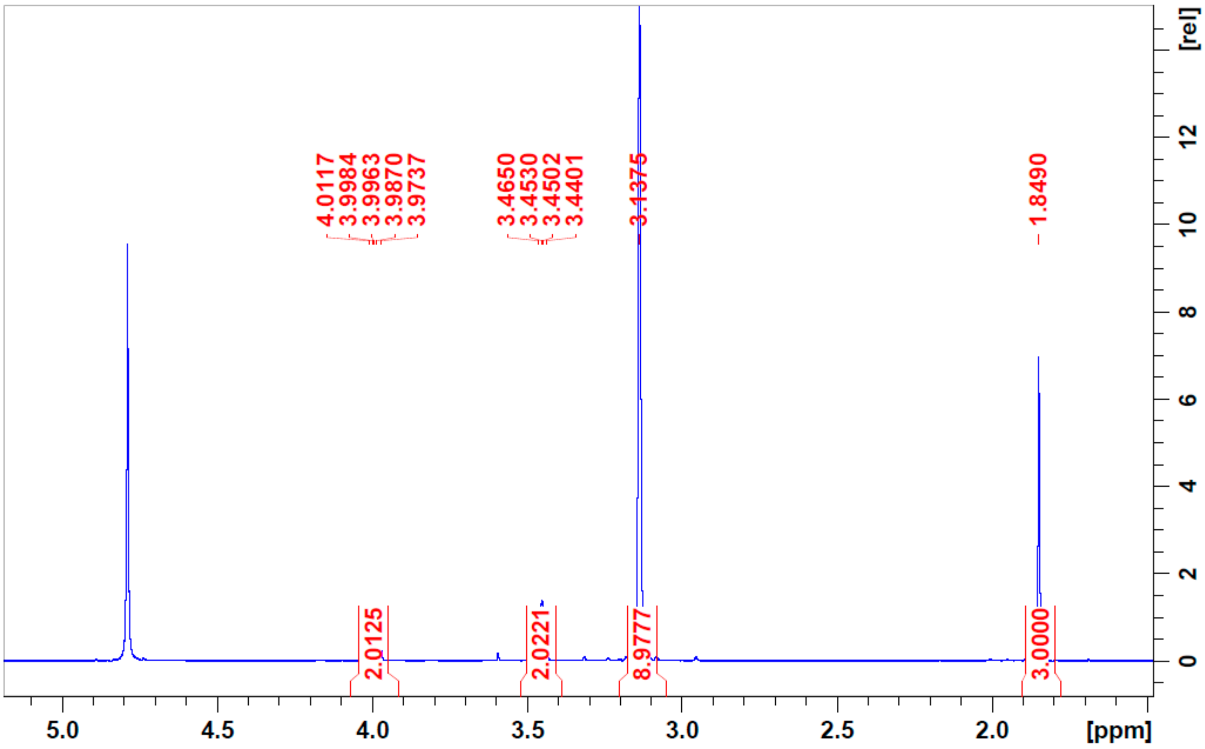

Figure S17. The ^{1}H NMR of [Ch][Ac].

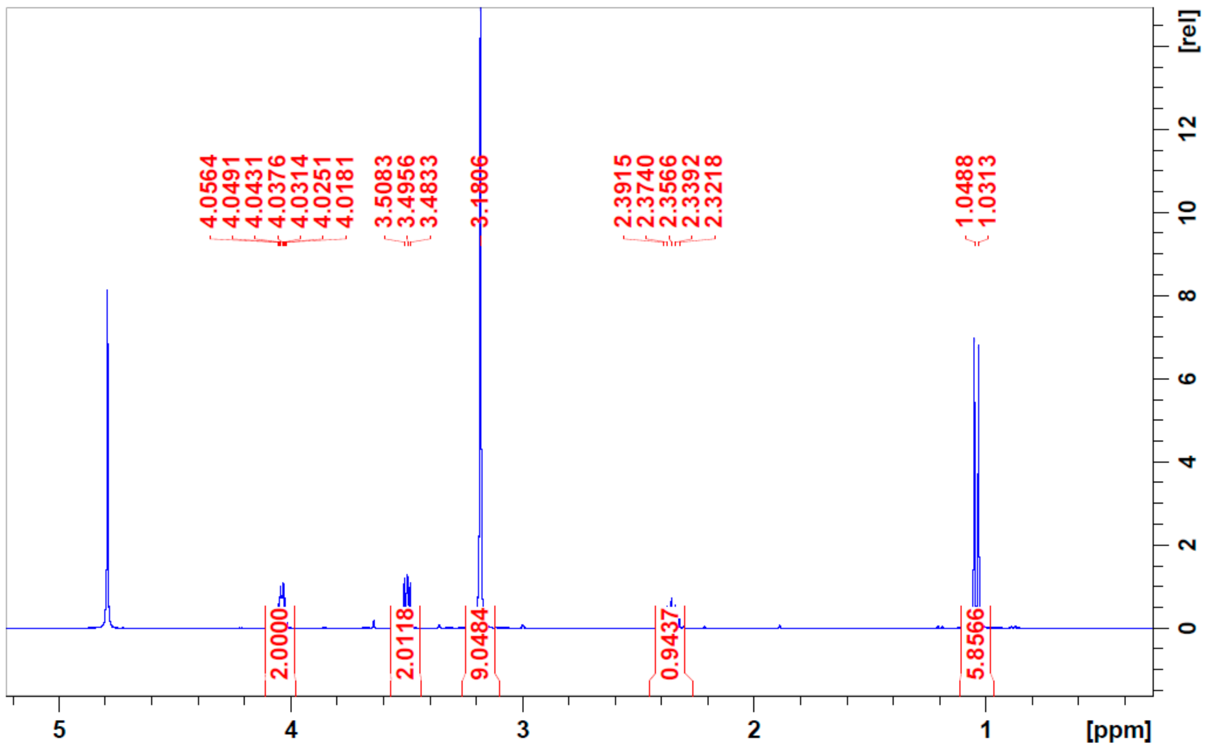

Figure S18. The ^{1}H NMR of [Ch][Ib].

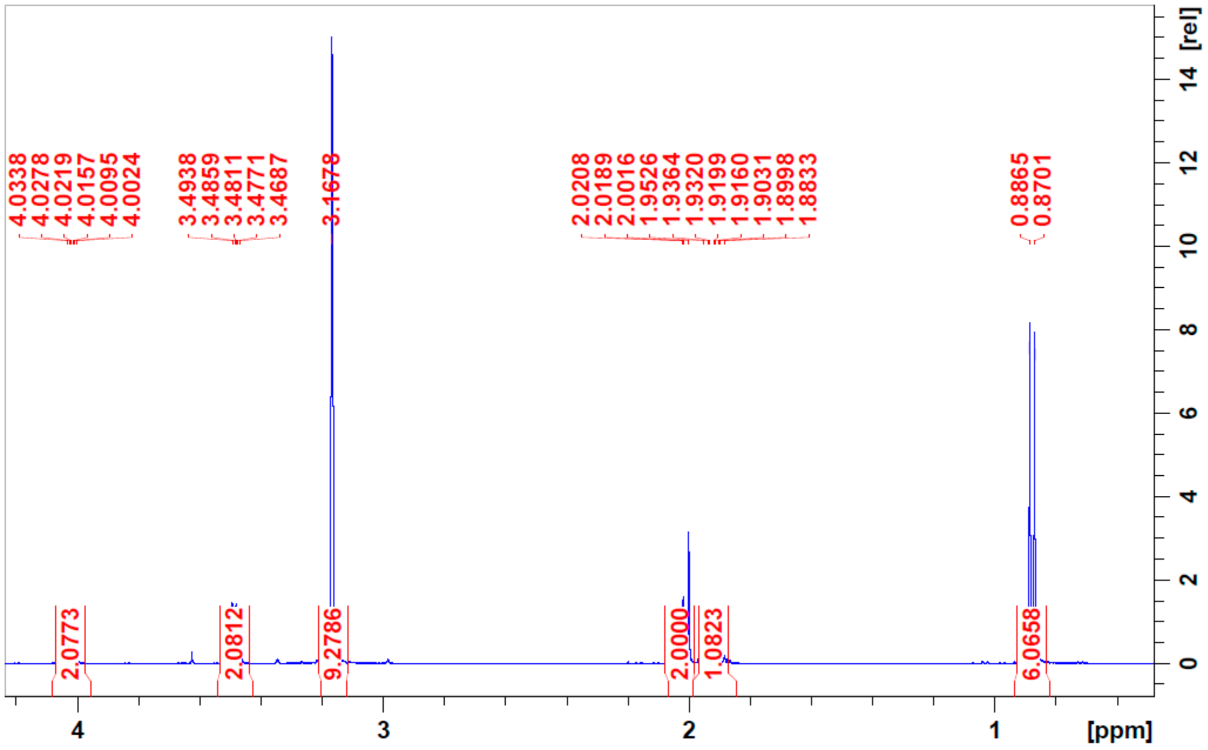

Figure S19. The ^{1}H NMR of [Ch][Iv].

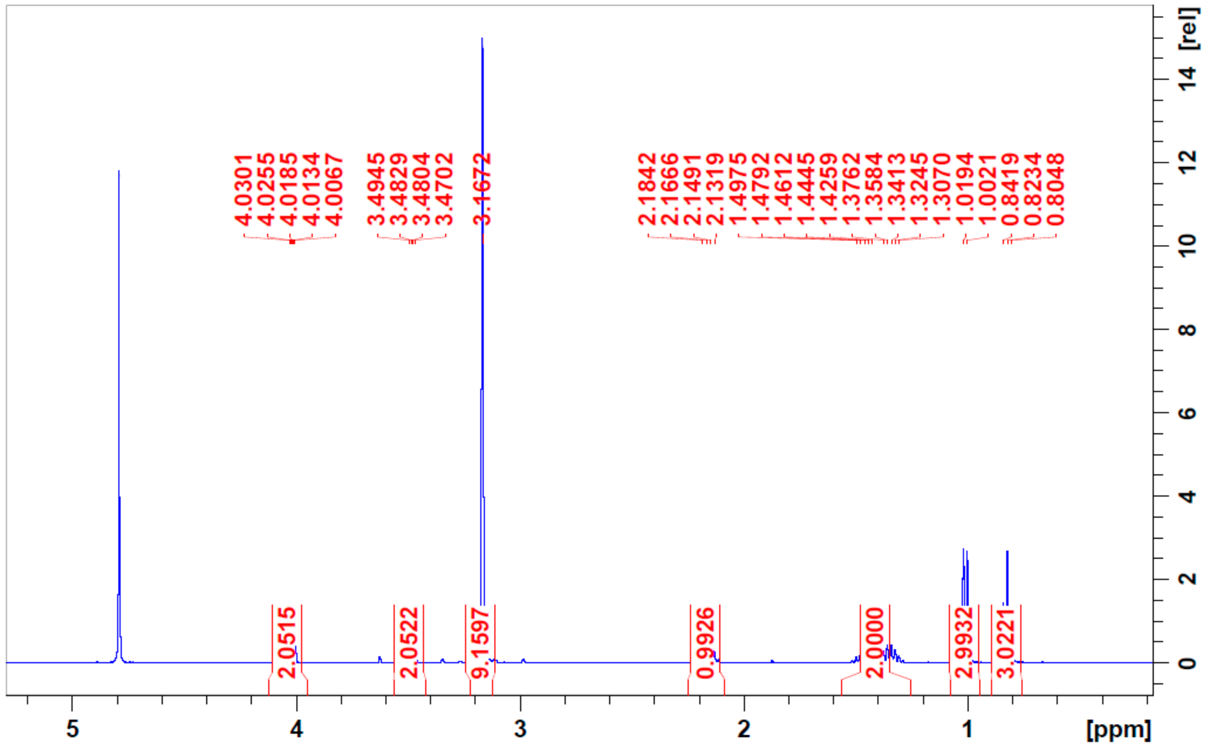

Figure S20. The ^{1}H NMR of [Ch][2mb].

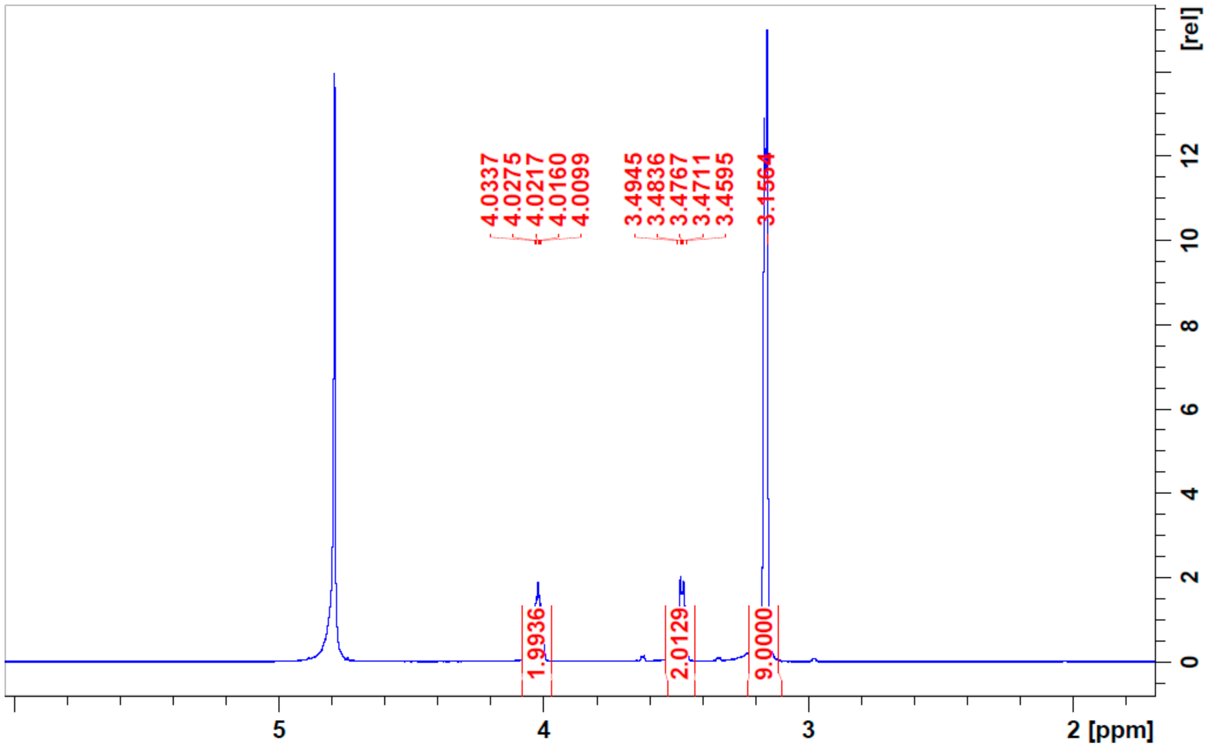

Figure S21. The ^{1}H NMR of [Ch][Mal].

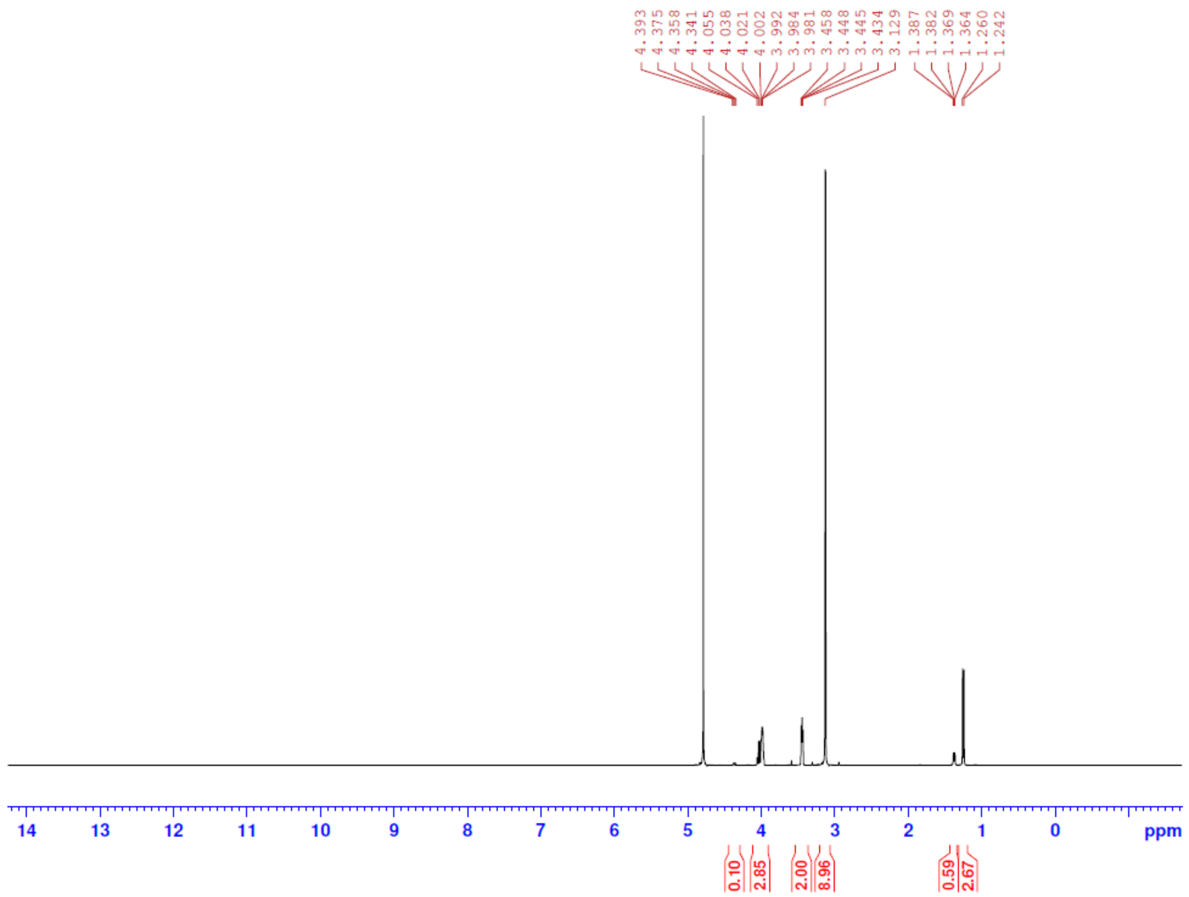

Figure S22. The ^{1}H NMR of [Ch][Lac].

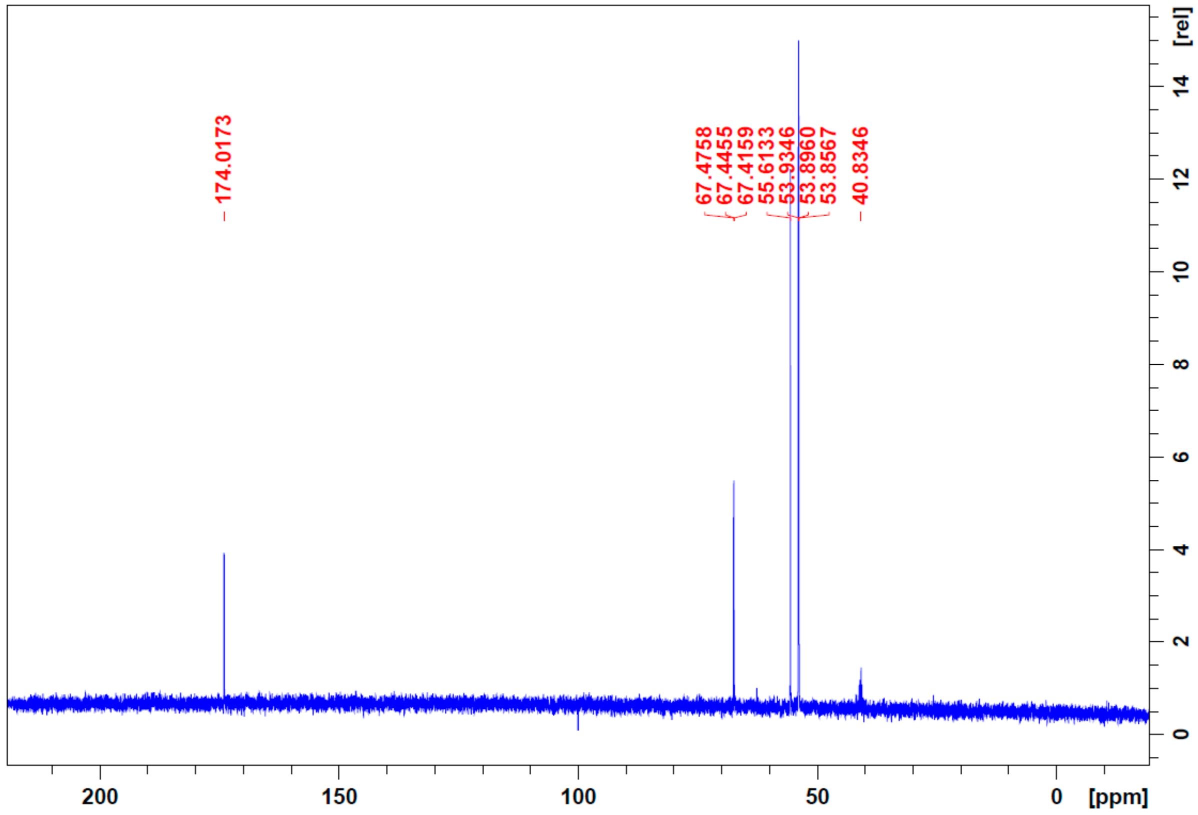

Figure S23. The ^{13}C NMR of [Ch][Mal].
